# ACFNet: Asymmetric Cross-Layer Fusion Network with Semantic Context Enhancement for Photovoltaic Defect Detection

**DOI:** 10.3390/ma19194249

**Published:** 2026-10-08

**Authors:** Li Zhang, Kaikai Chen, Huaping Guo, Zhuo Chen, Yange Sun, Hongzhou Yue, Yan Feng

**Affiliations:** 1 School of Computer and Information Technology, Xinyang Normal University, Xinyang 464000, China; zhangli@xynu.edu.cn (L.Z.); chenkaikai@xynu.edu.cn (K.C.); yangesun@xynu.edu.cn (Y.S.); yuehz@xynu.edu.cn (H.Y.); yfeng@xynu.edu.cn (Y.F.); 2 Henan Key Laboratory of Education Big Data Analysis and Application, Xinyang Normal University, Xinyang 464000, China; 3 School of Management and Economics, North China University of Water Resources and Electric Power, Zhengzhou 450045, China; chenzhuo1987@ncwu.edu.cn

**Keywords:** photovoltaic cell defect detection, object detection, attention mechanism, asymmetric feature fusion

## Abstract

Photovoltaic (PV) cell defects, such as cracks and star cracks, often arise during manufacturing and operation and may adversely affect the electrical performance and long-term reliability of photovoltaic cells. Detecting these defects in photovoltaic defect images is challenging due to complex background textures, scale variations, and structural discontinuity of defects. To address these challenges, we propose an asymmetric cross-layer fusion network (ACFNet) for photovoltaic cell defect detection. Specifically, we design a spatial-channel modulating block (SCMB) into the backbone to suppress background interference through joint spatial and channel modulation. We propose an asymmetric cross-layer fusion (ACF) strategy to handle scale variations by performing multiscale feature aggregation via asymmetric cross-layer connections. Furthermore, we develop a semantic context enhancer (SCE) in the detector heads to enhance the representation of discontinuous defect structures by combining large-kernel convolutions with parallel attention mechanisms. Experimental results on the PVEL-AD and PV-Multi-Defect datasets show that ACFNet achieves mAP@0.5 values of 0.933 and 0.884 and mAP@0.5:0.95 values of 0.652 and 0.598, respectively, with 3.0 M parameters and a measured throughput of 75.7 FPS on an NVIDIA A100 GPU, equivalent to an average processing time of approximately 13.2 ms per image.

## 1. Introduction

Photovoltaic (PV) cell defects, such as cracks, star cracks, and finger_interruption, frequently occur during manufacturing and operation [1,2]. Depending on their type and severity, these anomalies may adversely affect the electrical performance and long-term reliability of PV cells [3]. Achieving accurate defect detection in electroluminescence images is essential to ensure the reliability of PV systems [4], yet this task remains challenging due to complex background textures, substantial variations in defect size and morphology, and the structural discontinuity of crack-like defects.

Early photovoltaic defect detection methods mainly relied on handcrafted feature extraction combined with conventional machine learning algorithms for electroluminescence (EL) image analysis [5,6]. Although these methods are computationally efficient, their reliance on manually designed features limits their robustness and generalization under complex imaging conditions [7,8]. With the rapid development of deep learning, convolutional neural network (CNN)-based object detectors have become the dominant paradigm for PV defect detection [9]. Early CNN-based architectures, such as modified Faster R-CNN [10], improved defect localization accuracy through multi-stage feature extraction. To meet real-time industrial requirements, single-stage YOLO-based frameworks have been continuously optimized [11]. For instance, attention mechanisms were introduced to suppress background interference [12,13], while multiscale feature fusion strategies were developed to strengthen semantic interaction across different feature levels for irregular defects [14,15].

**Figure 1:**
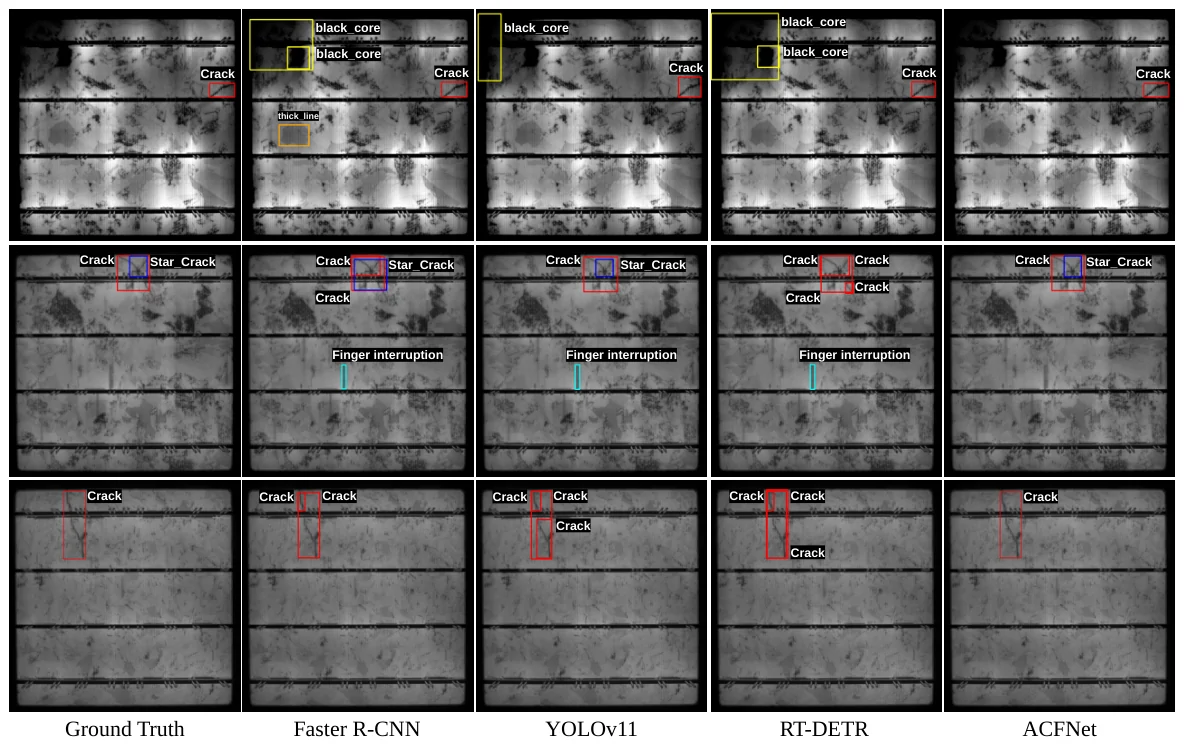
Visualization of detection results by four methods (Faster R-CNN, YOLOv11, RT-DETR, and ACFNet) alongside the Ground Truth on three challenging scenarios: complex background interference, significant scale variations, and defects with discontinuous structures.

Despite these advances, accurately detecting photovoltaic defects remains challenging due to complex background textures, scale variations, and structural discontinuity of defects. As illustrated in Figure 1, representative detectors, including Faster R-CNN [16], YOLOv11 [17], and RT-DETR [18], exhibit distinct limitations across these scenarios. For complex background interference (row 1), current detectors trigger redundant false positives in non-defective regions, frequently misidentifying background grain noise as black core or thick line defects. For significant scale variations (row 2), under the co-occurrence of star crack and crack defects, these methods suffer from category misclassification and false finger_interruption defect detections. For defects with discontinuous structures (row 3), these models generate redundant fragmented predictions along a single continuous crack.

To address these challenges, this paper proposes ACFNet, a novel asymmetric cross-layer fusion network for photovoltaic defect detection. Specifically, we design a plug-and-play spatial-channel modulating block (SCMB) integrated into the backbone to suppress complex background textures and purify feature representations via joint spatial gating and channel boosting. Furthermore, we propose an asymmetric cross-layer fusion (ACF) strategy in the neck to enhance feature pyramids via asymmetric skip connections, facilitating effective information exchange between high-resolution details and low-resolution semantics for defects with scale variations. In addition, we construct a semantic context enhancer (SCE) deployed in the detection heads to enhance the contextual representation of defect structures, enabling feature modeling for defects with discontinuous structures.

The main contributions of this paper are summarized as follows:

- We propose an ACFNet framework for photovoltaic defect detection, featuring an ACF strategy to facilitate multiscale feature fusion across high-resolution details and low-resolution semantics.
- We design an SCMB integrated into the backbone to suppress structural redundancy and purify feature representations through joint spatial gating and channel boosting.
- We construct an SCE deployed in the detection heads to enhance the contextual representation of defect structures, improving feature modeling for defects with discontinuous structures.

Extensive experiments on the PVEL-AD and PV-Multi-Defect datasets demonstrate that ACFNet achieves superior detection performance compared to existing state-of-the-art methods. The remainder of this paper is organized as follows. Section 2 reviews the related work. Section 3 introduces the proposed ACFNet and its key modules. Section 4 presents the experimental results, including comparisons with state-of-the-art methods and ablation analyses. Finally, Section 5 concludes this paper.

## 2. Related Work

### 2.1. Evolution of Photovoltaic Cell Defect Detection

Early photovoltaic defect detection relied primarily on traditional machine learning and image processing approaches. Representative methods include handcrafted feature extraction with support vector machines (SVMs) [5], spectrum-analysis-based periodic feature reconstruction for defect detection [19], and conventional machine learning classifiers for defect identification [6]. However, these approaches depend heavily on handcrafted features and manually designed processing pipelines, limiting their robustness against background noise, low contrast, and non-uniform defect morphologies in EL images [8].

To overcome these constraints, deep learning-based object detectors have become the mainstream paradigm, broadly categorized into CNN-based architectures and Transformer-based models. Within CNN-based approaches, researchers have tailored various feature enhancement mechanisms to tackle EL image challenges. To mitigate feature vanishing and improve multiscale defect localization, bidirectional attention pyramids have been integrated into multi-stage architectures [20]. To combat intense background noise, specialized feature suppression and spatial modulation mechanisms were introduced to clean the representation space [21]. Meanwhile, lightweight structural redesigns and practical benchmarkings have been explored to meet the demands of real-time industrial inspection [13,22]. Building on these foundations, Vision Transformers and DETR-based paradigms have recently emerged to capture long-range dependencies. To model global contextual relationships, hybrid architectures embedding Transformer modules within CNNs have been explored [23]. Furthermore, to achieve superior accuracy while maintaining real-time execution in challenging industrial environments, customized DETR-based frameworks have been proposed [24].

### 2.2. Multiscale Representation Learning

Multiscale representation learning in photovoltaic defect detection relies on feature pyramid structures, such as FPN [25], PANet [26], and BiFPN [27], to merge high-level semantic information with low-level spatial details across different resolutions. GiraffeDet further extends multiscale feature interaction through a GFPN with dense connections across different spatial scales and semantic levels [28]. To address severe scale variations and complex background noise in EL images, recent approaches integrate attention modules and neighborhood aggregation to refine multiscale feature selection [14,29,30]. Meanwhile, other methods introduce multiscale edge operators, coordinate-aware blocks, and deformable convolutions to better capture irregular defect morphologies [15,31,32,33].

Despite these advances, existing feature pyramid strategies often fail to balance high-resolution fine details with low-resolution global semantics in PV defect detection. Standard symmetric cross-scale fusion tends to dilute delicate spatial boundaries while blurring high-level contextual features during progressive aggregation. Consequently, current feature pyramids remain inadequate for handling extreme scale variations and structural discontinuities in EL images, necessitating asymmetric fusion and contextual feature enhancement.

## 3. Methodology

### 3.1. Overall Architecture

**Figure 2:**
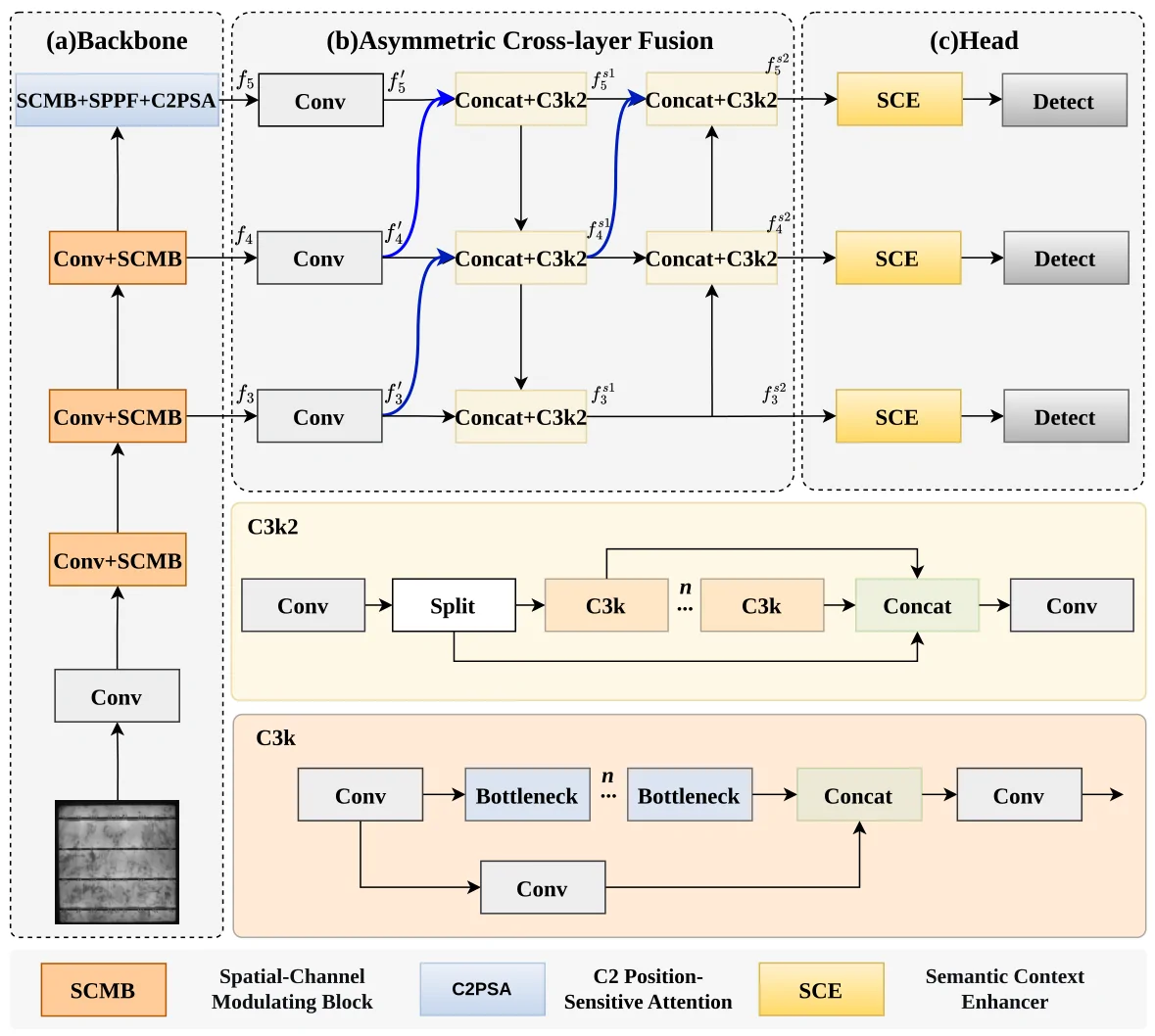
Overall architecture of the proposed ACFNet, comprising a feature extractor integrated with SCMBs, an ACF strategy, and detection heads equipped with SCE.

Figure 2 illustrates the overall architecture of ACFNet, consisting of three main components: an SCMB-augmented backbone, an ACF neck, and an SCE-enhanced detection head. We adopt a CSPNet [34] to extract multiscale features $\left\{f_{3},f_{4},f_{5}\right\}$ due to its strong representation capability and high computational efficiency. In addition, we design a plug-and-play SCMB and integrate it into CSPNet to suppress complex background interference and eliminate feature redundancy, enhancing the feature extraction capability for fine-grained defect cues. Formally,

(1) $$f_{3},f_{4},f_{5}=F_{\mathrm{Backbone}}\left(X\right),$$

where $X\in R^{H\times W\times C}$ is an input PV defect image.

The multiscale features $\left\{f_{3},f_{4},f_{5}\right\}$ are initially harmonized via channel-adjusting convolutions to yield $\left\{f_{3}^{\prime },f_{4}^{\prime },f_{5}^{\prime }\right\}$, which are subsequently fed into the ACF neck. Unlike conventional symmetric feature pyramids, ACF introduces asymmetric cross-layer skip connections and cross-scale aggregation paths:

(2) $$f_{3}^{s_{2}},f_{4}^{s_{2}},f_{5}^{s_{2}}=F_{\mathrm{ACF}}\left(f_{3}^{\prime },f_{4}^{\prime },f_{5}^{\prime }\right),$$

where $F_{\mathrm{ACF}}\left(\cdot \right)$ denotes the asymmetric cross-layer fusion operation that establishes direct interaction between non-adjacent layers, bridging high-resolution spatial details with abstract semantic contextual features without information loss.

Finally, the fused features $\left\{f_{3}^{s_{2}},f_{4}^{s_{2}},f_{5}^{s_{2}}\right\}$ are refined by the SCE modules and then fed into the detection heads to generate the final classification scores and bounding box predictions. By refining contextual features prior to final inference, SCE enhances the feature representation of defects with discontinuous structures across all prediction scales. The main contributions of our ACFNet lie in SCMB, ACF and SCE, and the corresponding details are presented in Section 3.2, Section 3.3 and Section 3.4.

### 3.2. Spatial-Channel Modulating Block (SCMB)

**Figure 3:**
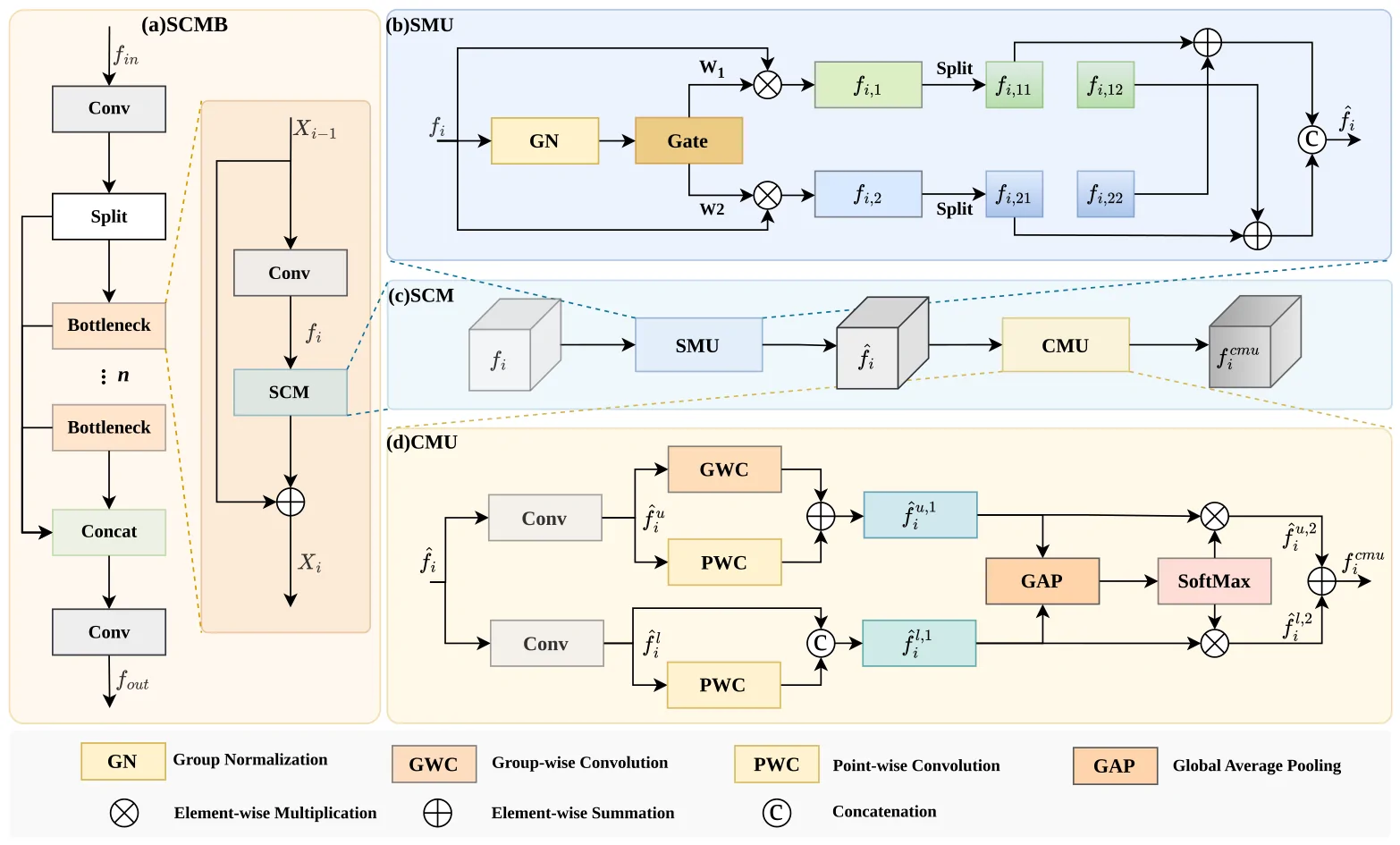
Architecture of the SCMB. (**a**) Overall structure of the spatial-channel modulating block (SCMB). (**b**) Spatial Modulation Unit (SMU). (**c**) Spatial-Channel Modulator (SCM). (**d**) Channel Modulation Unit (CMU).

In PV defect images, weak defect signatures are easily overwhelmed and obscured by complex background textures, leading standard backbones to confuse subtle anomalies with noise and suppress critical defect cues. To address this challenge, we propose SCMB as a plug-in module that dynamically filters background-dominated activations and recalibrates feature responses through a C3k2-based split-and-aggregate architecture, in which the sequential SMU–CMU modulation is embedded within residual Bottlenecks, while drawing on related spatial and channel modulation principles from ScConv [35], as shown in Figure 3.

As illustrated in Figure 3a, SCMB adopts a split-and-aggregate design to process multiscale features. Given an input feature $f_{\mathrm{in}}$, SCMB first applies a 1×1 convolution, followed by a channel-wise split operation to partition the feature into two sub-branches:

(3) $$f_{sub1},f_{sub2}=\mathrm{Split}\left(\mathrm{Conv}(f_{\mathrm{in}})\right).$$

The primary branch $f_{sub1}$ passes through *n* sequentially stacked Bottleneck units, where each unit embeds a spatial-channel modulator (SCM) in a residual structure. Formally, for the *i*-th Bottleneck, the output is computed as:

(4) $$X_{i}=X_{i-1}+F_{\mathrm{SCM}}\left(\mathrm{Conv}(X_{i-1})\right),\;i\in \left\{2,\ldots ,n\right\}$$

where $X_{1}=f_{sub1}$ and $F_{\mathrm{SCM}}\left(\cdot \right)$ denotes the sequential spatial-then-channel modulation operation executing SMU followed by CMU (shown in Figure 3c):

(5) $$f_{i}^{\mathrm{cmu}}=F_{\mathrm{SCM}}\left(f_{i}\right)=\mathrm{CMU}\left(\mathrm{SMU}(f_{i})\right).$$

Finally, the output from the *n*-th Bottleneck $X_{n}$ is concatenated with the retained identity branch $f_{sub2}$ and processed through a concluding convolution layer to yield the refined output feature $f_{\mathrm{out}}$:

(6) $$f_{\mathrm{out}}=\mathrm{Conv}\left(\mathrm{Concat}(X_{n},f_{sub2})\right).$$

#### 3.2.1. Spatial Modulation Unit (SMU)

To mitigate spatial noise interference and preserve subtle defect structures, we design SMU to separate background-dominated activations from informative regions and reconstruct their spatial interactions.

As illustrated in Figure 3b, given an input feature $f_{i}\in R^{H\times W\times C}$, Group Normalization (GN) [36] is first applied to obtain normalized features. A hard gating mechanism with a fixed threshold τ (e.g., τ=0.5) is then employed to partition high-activation regions from weak ones:

(7) $$W_{1}=I\left(\sigma \left(\mathrm{GN}(f_{i})\right)\geq \tau \right),\;W_{2}=1-W_{1},$$

where σ(·) denotes the Sigmoid activation function, and I(·) represents the indicator function yielding binary spatial masks. The input feature $f_{i}$ is modulated by $W_{1}$ and $W_{2}$ to form two complementary spatial feature groups:

(8) $$f_{i,1}=W_{1}⊗f_{i},\;f_{i,2}=W_{2}⊗f_{i},$$

where ⊗ denotes element-wise multiplication.

Subsequently, SMU adopts a cross-reconstruction strategy to merge $f_{i,1}$ and $f_{i,2}$, establishing spatial continuity across complementary response regions. Specifically, $f_{i,1}$ and $f_{i,2}$ are evenly split along the channel dimension into sub-features $\left(f_{i,11},f_{i,12}\right)$ and $\left(f_{i,21},f_{i,22}\right)$, respectively. These sub-features are cross-aggregated and concatenated as:

(9) $${\hat{f}}_{i}=\mathrm{Concat}\left(f_{i,11}⊕f_{i,22};f_{i,12}⊕f_{i,21}\right),$$

where ⊕ denotes element-wise summation and ${\hat{f}}_{i}$ serves as the spatially recalibrated feature representation for subsequent channel-wise modulation.

#### 3.2.2. Channel Modulation Unit (CMU)

To complement spatial modulation of SMU, we design CMU to adaptively recalibrate channel-wise responses via dual transformation branches. As illustrated in Figure 3d, CMU first splits the spatially modulated feature ${\hat{f}}_{i}\in R^{H\times W\times C}$ derived from SMU along the channel dimension into two parallel sub-branches, each with *C* channels. A 1×1 convolution is applied to each sub-branch to generate intermediate representations ${\hat{f}}_{i}^{u}$ and ${\hat{f}}_{i}^{l}$.

The upper sub-branch processes ${\hat{f}}_{i}^{u}$ through group-wise convolution (GWC) and point-wise convolution (PWC) in parallel, and fuses their outputs via element-wise addition:

(10) $${\hat{f}}_{i}^{u,1}=\mathrm{GWC}\left({\hat{f}}_{i}^{u}\right)⊕\mathrm{PWC}\left({\hat{f}}_{i}^{u}\right).$$

Unlike the upper sub-branch, the lower sub-branch only applies a PWC to ${\hat{f}}_{i}^{l}$, followed by a shortcut connection to retain the original information:

(11) $${\hat{f}}_{i}^{l,1}=\mathrm{Concat}\left(\mathrm{PWC}\left({\hat{f}}_{i}^{l}\right),{\hat{f}}_{i}^{l}\right).$$

Subsequently, ${\hat{f}}_{i}^{u,1}$ and ${\hat{f}}_{i}^{l,1}$ are concatenated along the channel dimension. Global average pooling (GAP) and SoftMax are sequentially applied to generate normalized channel weights:

(12) $$\beta =\mathrm{SoftMax}\left(\mathrm{GAP}\left(\mathrm{Concat}({\hat{f}}_{i}^{u,1},{\hat{f}}_{i}^{l,1})\right)\right),$$

where $\beta =[\beta_{u},\beta_{l}]$ represents the attention weights split according to the channel dimensions of the upper and lower sub-branches. Finally, the recalibrated features are fused via element-wise summation to produce the modulated output:

(13) $$f_{i}^{\mathrm{cmu}}=\left(\beta_{u}⊗{\hat{f}}_{i}^{u,1}\right)⊕\left(\beta_{l}⊗{\hat{f}}_{i}^{l,1}\right).$$

### 3.3. Asymmetric Cross-Layer Fusion (ACF)

Conventional feature pyramids rely on sequential adjacent-layer propagation, which often attenuates cross-scale interactions in the presence of scale variations and structural discontinuities. To address this limitation, we propose the ACF strategy to facilitate direct information flow between semantic and structural representations.

As illustrated in Figure 2b, ACF operates on three multiscale features extracted from the backbone, denoted as $F=\{f_{3},f_{4},f_{5}\}$. First, a 1×1 convolution is applied to each scale to unify the channel dimensions:

(14) $$f_{i}^{\prime }={\mathrm{Conv}}_{1\times 1}\left(f_{i}\right),\;i\in 3,4,5.$$

To enable dense cross-scale information exchange, ACF employs a two-stage recursive aggregation mechanism. In the first stage, a top-down pathway combines features from adjacent and non-adjacent scales:

(15) $$f_{i}^{s_{1}}=C3k2\left(\left[I\left(i<5\right)\;U\left(f_{i+1}^{s_{1}}\right);f_{i}^{\prime };I\left(i>3\right)D\left(f_{i-1}^{\prime }\right)\right]\right),\;i\in \left\{3,4,5\right\},$$

where U(·) and D(·) denote upsampling and downsampling operations for spatial alignment, respectively, and I(·) represents the indicator function.

In the second stage, a bottom-up pathway complements deep layers with rich structural details to generate the final feature representations $\left\{f_{3}^{s_{2}},f_{4}^{s_{2}},f_{5}^{s_{2}}\right\}$:

(16) $$f_{i}^{s_{2}}=\left\{\begin{array}{cc} f_{3}^{s_{1}}, & i=3,\\ C3k2\left(\left[f_{i}^{s_{1}};D\left(f_{i-1}^{s_{2}}\right);I\left(i=5\right)D\left(f_{4}^{s_{1}}\right)\right]\right), & i\in \{4,5\}. \end{array}\right.$$

Here, the additional term $I\left(i=5\right)D\left(f_{4}^{s_{1}}\right)$ introduces a direct cross-stage path from the intermediate representation $f_{4}^{s_{1}}$ to the deepest node $f_{5}^{s_{2}}$, reinforcing the semantic feature with multi-path contextual information.

### 3.4. Semantic Context Enhancer (SCE)

**Figure 4:**
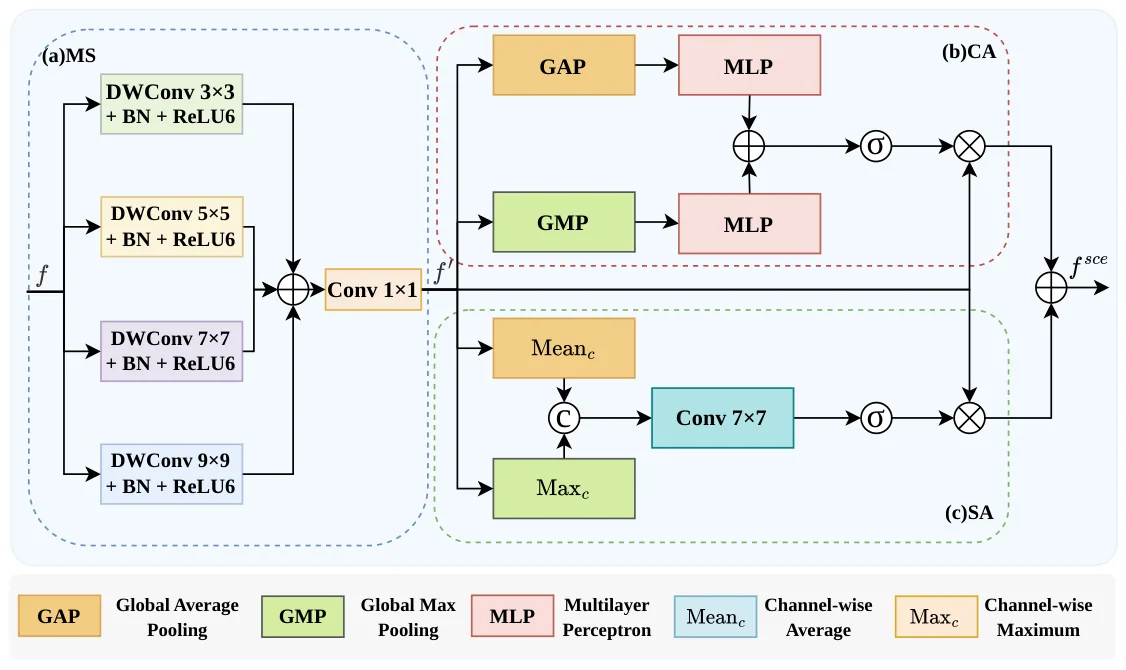
Architecture of the SCE module.

To enhance the representation of defects with discontinuous structures, we propose the SCE before the detection heads to refine multiscale representations. As illustrated in Figure 4, SCE consists of three main sub-modules: a multiscale semantic (MS) module, a channel attention (CA) branch, and a spatial attention (SA) branch. Formally, given an input feature $f\in \{f_{3}^{s_{2}},f_{4}^{s_{2}},f_{5}^{s_{2}}\}$, the overall pipeline of SCE is formulated as:

(17) $$f^{\prime }=\mathrm{MS}\left(f\right),$$

(18) $$f^{\mathrm{sce}}=\mathrm{CA}\left(f^{\prime }\right)⊕\mathrm{SA}\left(f^{\prime }\right).$$

MS captures multi-receptive-field contextual information through four parallel depthwise convolution branches with kernel sizes k∈{3,5,7,9}. Each branch is followed by batch normalization (BN) and ReLU6 activation, and the resulting features are aggregated by element-wise summation and further projected through a 1×1 convolution:

(19) $$\begin{array}{c} \mathrm{MS}\left(f\right)={\mathrm{Conv}}_{1\times 1}(\underset{k\in \{3,5,7,9\}}{⨁}\mathrm{ReLU}6\left(\mathrm{BN}\left({\mathrm{DWConv}}_{k}\left(f\right)\right)\right)). \end{array}$$

where ⨁ denotes element-wise summation, and the output of the 1×1 convolution is denoted as the aggregated context feature $f^{\prime }$.

To suppress redundant background noise while highlighting defect regions, $f^{\prime }$ is fed into parallel CA and SA branches. The CA and SA branches adopt pooling-based attention operations similar to CBAM [37]. The CA branch recalibrates channel-wise responses via global average pooling (GAP) and global max pooling (GMP):

(20) $$\mathrm{CA}\left(f^{\prime }\right)=f^{\prime }⊗\sigma \left(\mathrm{MLP}\left(\mathrm{GAP}\left(f^{\prime }\right)\right)⊕\mathrm{MLP}\left(\mathrm{GMP}\left(f^{\prime }\right)\right)\right),$$

where σ(·) denotes the sigmoid activation function, and ⊗ represents element-wise multiplication. Concurrently, the SA branch highlights structurally important spatial regions:

(21) $$\mathrm{SA}\left(f^{\prime }\right)=f^{\prime }⊗\sigma \left({\mathrm{Conv}}_{7\times 7}\left(\mathrm{Concat}\left({\mathrm{Mean}}_{c}\left(f^{\prime }\right),{\mathrm{Max}}_{c}\left(f^{\prime }\right)\right)\right)\right).$$

The outputs of the CA and SA branches are finally fused by element-wise summation to produce the refined feature representation:

(22) $$f^{\mathrm{sce}}=\mathrm{CA}\left(f^{\prime }\right)⊕\mathrm{SA}\left(f^{\prime }\right),$$

where ⊕ denotes element-wise summation.

## 4. Experiments

### 4.1. Experimental Setting

#### 4.1.1. Datasets

Experiments are conducted on two public photovoltaic defect datasets: PVEL-AD [38] and PV-Multi-Defect [39]. PVEL-AD contains 36,543 EL images covering 12 defect categories. Representative defect types may have different physical origins, with crack-like defects commonly associated with mechanical or thermomechanical stress and finger_interruptions related to metallization degradation [40,41]. Following standard practices in the photovoltaic defect-detection literature, our evaluation on PVEL-AD focuses on seven representative defect categories: black_core (Bc), crack (Cr), finger_interruption (Fi), horizontal_dislocation (Hd), short_circuit (Sci), star_crack (Scr), and thick_line (Tl). Our evaluation uses 4975 annotated images covering these seven categories. From an industrial quality-control (QC) perspective, these categories cover representative defect modes routinely assessed in EL-based inspection because of their potential effects on cell performance and module reliability [40,42]. Some excluded categories account for less than 1% of all annotated instances in this public benchmark and are therefore severely underrepresented. Vertical_dislocation was also excluded because it is an underrepresented orientation-specific variant of the dislocation pattern already represented by horizontal_dislocation. Industrial PV inspection studies show that severe class imbalance biases detectors toward majority classes and limits their recognition of minority defects, thereby reducing the reliability of automated quality-control decisions [43].

**Figure 5:**
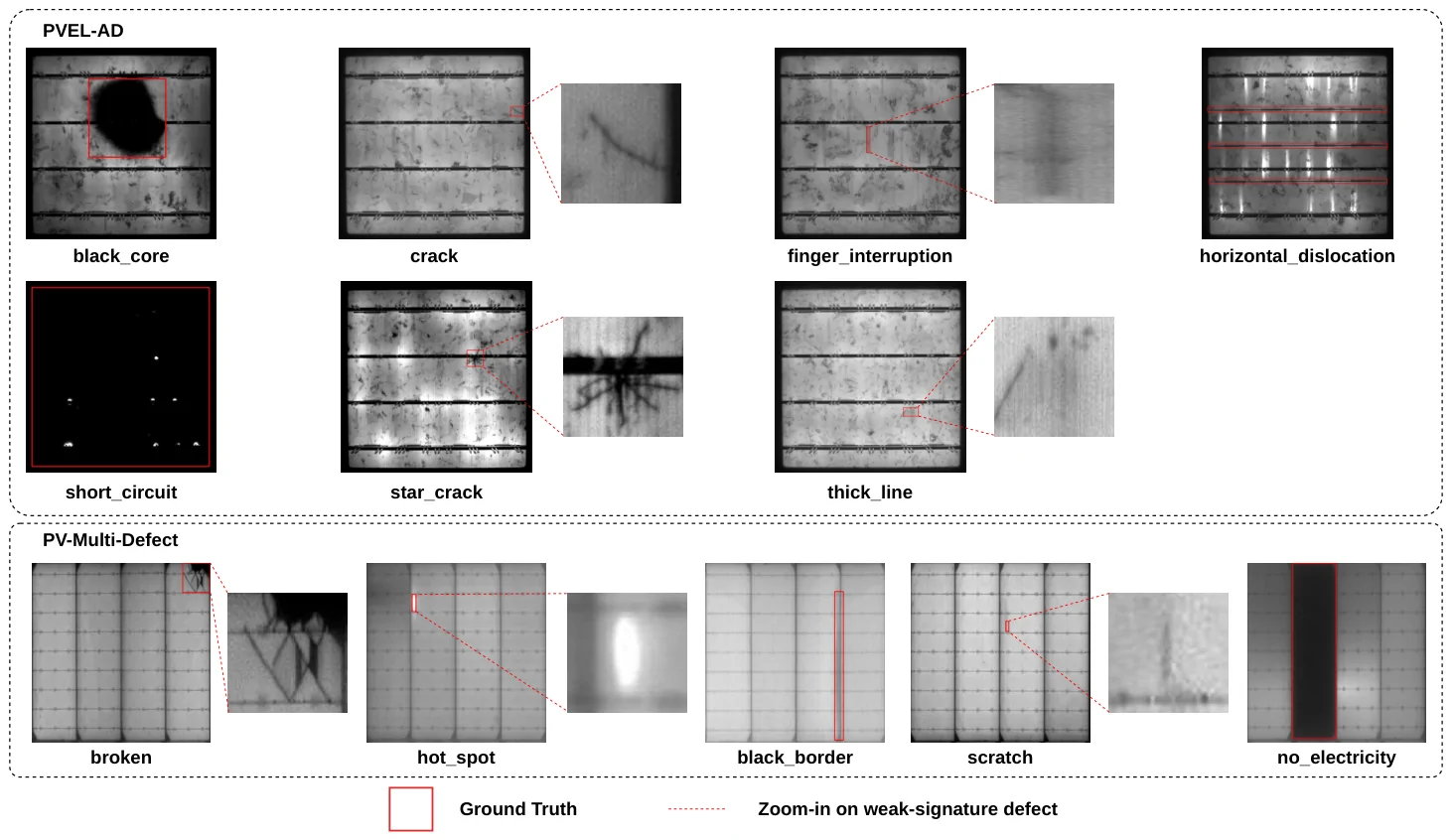
Representative ground-truthed image and zoomed-in views of defect classes in the PVEL-AD and PV-Multi-Defect datasets. Red boxes indicate ground-truth annotations, and zoomed-in views highlight weak-signature defect patterns.

PV-Multi-Defect contains 1108 camera-acquired surface images with five defect categories, namely broken (Br), hot_spot (Hs), black_border (Bb), scratch (Sc), and no_electricity (Ne). As illustrated in Figure 5, representative samples with ground-truth annotations cover diverse morphological characteristics across both datasets. The corresponding zoomed-in views highlight the subtle structural details and weak visual signatures typical of fine-grained PV defects. Following the established literature [44], both datasets are randomly partitioned into mutually exclusive training, validation, and test sets at an 8:1:1 ratio. This standardized setup ensures consistent evaluation conditions and enables fair performance comparisons with SOTA baselines.

#### 4.1.2. Evaluation Metrics

We adopt Precision (*P*), Recall (*R*), the $F_{1}$ score, Average Precision (AP), mAP@0.5, and mAP@0.5:0.95 [45] to evaluate the performance of our ACFNet. Let TP, FP, and FN denote the number of true positives, false positives, and false negatives, respectively. The metrics *P*, *R*, and $F_{1}$ are defined as:

(23) $$P=\frac{TP}{TP+FP},\;R=\frac{TP}{TP+FN},\;F_{1}=\frac{2\times P\times R}{P+R}.$$

To account for varying confidence thresholds, AP computes the integral area under the precision–recall curve:

(24) $$AP=\int_{0}^{1}P\left(R\right)\;dR.$$

mAP@0.5 denotes the mean AP across all defect categories at an IoU threshold of 0.50, whereas mAP@0.5:0.95 denotes the mean AP averaged over IoU thresholds from 0.50 to 0.95 with a step of 0.05. The corresponding class-wise metrics are denoted as AP@0.5 and AP@0.5:0.95. Precision, Recall, and F1-score are reported at an IoU threshold of 0.50 and at the confidence threshold maximizing each model’s smoothed mean F1 curve across classes. AP is computed from confidence-ranked detections using the respective frameworks’ native evaluation implementations with 101-point recall interpolation.

#### 4.1.3. Implementation Details

Our ACFNet is implemented using the PyTorch 2.7.1 and trained on an NVIDIA A100 GPU with 80 GB memory. All input images are resized to 640×640 for both training and testing. The model is trained for 300 epochs with a batch size of 16. Stochastic Gradient Descent (SGD) serves as the optimizer with an initial learning rate of 0.01. The Complete IoU (CIoU) loss [46] is adopted as the bounding-box regression loss during training. In an automated inspection setup, ACFNet functions as a software vision inference module that receives EL images and outputs structured metadata (defect categories and bounding box coordinates) to serve downstream PLC sorting decisions and MES quality traceability.

### 4.2. Comparison with SOTA Methods

#### 4.2.1. Quantitative Comparison

To evaluate the performance of our ACFNet, we select twenty-one methods for comparison, including three two-stage detectors (Faster R-CNN [16], Cascade R-CNN [47], and Dynamic R-CNN [48]), nine one-stage and anchor-free detectors (RetinaNet [49], FCOS [50], ATSS [51], YOLOv8 [52], YOLOv11 [17], YOLOv12 [53], YOLO26 [54], YOLOX [55], and RTMDet [56]), six Transformer-based detectors (DETR [57], Deformable DETR [58], Conditional DETR [59], DAB-DETR [60], DINO [61], and RT-DETR [18]); and three recent specialized PV defect detectors (PSDD [21], AE-YOLO [13], and YOLO-PV [29]). The results of comparing methods are obtained by running official codes with default parameter settings. Table 1 and Table 2 show the corresponding results on the PVEL-AD and PV-Multi-Defect datasets, respectively.

As shown in Table 1, ACFNet achieves the highest mAP@0.5, Precision, Recall, and F1-score, with values of 0.933, 0.907, 0.890, and 0.898, respectively, while obtaining an mAP@0.5:0.95 of 0.652. Compared with RTMDet, ACFNet increases the mAP@0.5 by 0.02. Compared with the specialized PV defect detectors PSDD, AE-YOLO, and YOLO-PV, the mAP@0.5 of ACFNet is higher by 0.009, 0.016, and 0.021, respectively. For specific defect categories, ACFNet outperforms all competing methods in four key classes, achieving the highest AP@0.5 values in crack (Cr, 0.821), finger_interruption (Fi, 0.921), star crack (Scr, 0.907), and thick line (Tl, 0.907). In the remaining categories (Bc, Hd, and Sci), although ACFNet is marginally lower than the top-performing models (e.g., ATSS in Bc and Hd by 0.008 and 0.002, and Dynamic R-CNN/RTMDet in Sci by 0.007), it consistently maintains near-optimal performance (>0.990).

From Table 2, ACFNet achieves the highest overall performance on the PV-Multi-Defect dataset, obtaining an mAP@0.5 of 0.884 and an mAP@0.5:0.95 of 0.598, Precision of 0.878, Recall of 0.822, and an F1 score of 0.849. Compared with the second-best model DINO, ACFNet improves the mAP@0.5 by 0.024. In terms of category-specific AP@0.5, ACFNet achieves peak performance in broken (Br, 0.922) and black border (Bb, 0.987), outperforming the second-best models (YOLO-PV and PSDD) by 0.005 and 0.009, respectively. In other categories, although ACFNet yields lower AP@0.5 values than specific baselines in hot spot (Hs, behind RetinaNet/Deformable DETR by 0.074), scratch (Sc, behind FCOS by 0.056), and no electricity (Ne, behind RetinaNet by 0.062), its balanced feature representations across all defect types lead to the highest overall mAP@0.5. Nevertheless, ACFNet performs less well than some competing methods on certain defect categories, including hot spots and scratches. This may be related to their weak or localized visual signatures and susceptibility to background interference. Feature fusion may strengthen weak defect responses, but it may also amplify background-related responses, creating a potential precision–recall trade-off between detecting subtle defects and suppressing false positives.

From an industrial quality assurance perspective, the high Precision and Recall metrics across both benchmarks carry direct operational significance. On PVEL-AD (Table 1), ACFNet achieves a Precision of 0.907 and a Recall of 0.890, where high Precision effectively minimizes false rejects by preventing the unnecessary scrapping of defect-free modules, and high Recall strictly caps false accepts by suppressing critical defect escapes. This balanced decision capability generalizes consistently to PV-Multi-Defect (Table 2) with a Precision of 0.878 and a Recall of 0.822, confirming its operational stability across varying inspection environments. As a supplementary evaluation, ACFNet was tested on 11,353 anomaly-free images from the official PVEL-AD release at a confidence threshold of 0.5. It produced 130 false detections in 129 images, yielding an FPPI (false positives per image) of 0.0115 and an image-level specificity (percentage of images without false detections) of 98.86%.

#### 4.2.2. Statistical Significance Analysis

To assess the stability and statistical significance of the performance gains, ACFNet and the strongest competing method on each dataset (PSDD on PVEL-AD and DINO on PV-Multi-Defect) are evaluated across five fixed random seeds. As reported in Table 3, ACFNet consistently achieves strong mean performance on key detection metrics across different random seeds. Specifically, ACFNet achieves the highest mAP@0.5 of 0.934±0.003 on PVEL-AD and 0.876±0.006 on PV-Multi-Defect. Furthermore, a paired *t*-test confirms that the mAP@0.5 improvement over PSDD on PVEL-AD is statistically significant (p<0.05). These results demonstrate the stability of ACFNet across different random seeds, with a statistically significant mAP@0.5 improvement on PVEL-AD.

As shown in Table 4, image-level paired bootstrap analysis yields an mAP@0.5 difference of 0.015 between ACFNet and PSDD on PVEL-AD, with a 95% confidence interval of [0.002,0.029], indicating a positive difference that does not include zero. On PV-Multi-Defect, the mAP@0.5 difference between ACFNet and DINO is also 0.015, while the corresponding 95% confidence interval of [−0.015,0.046] includes zero.

#### 4.2.3. Qualitative Comparison

**Figure 6:**
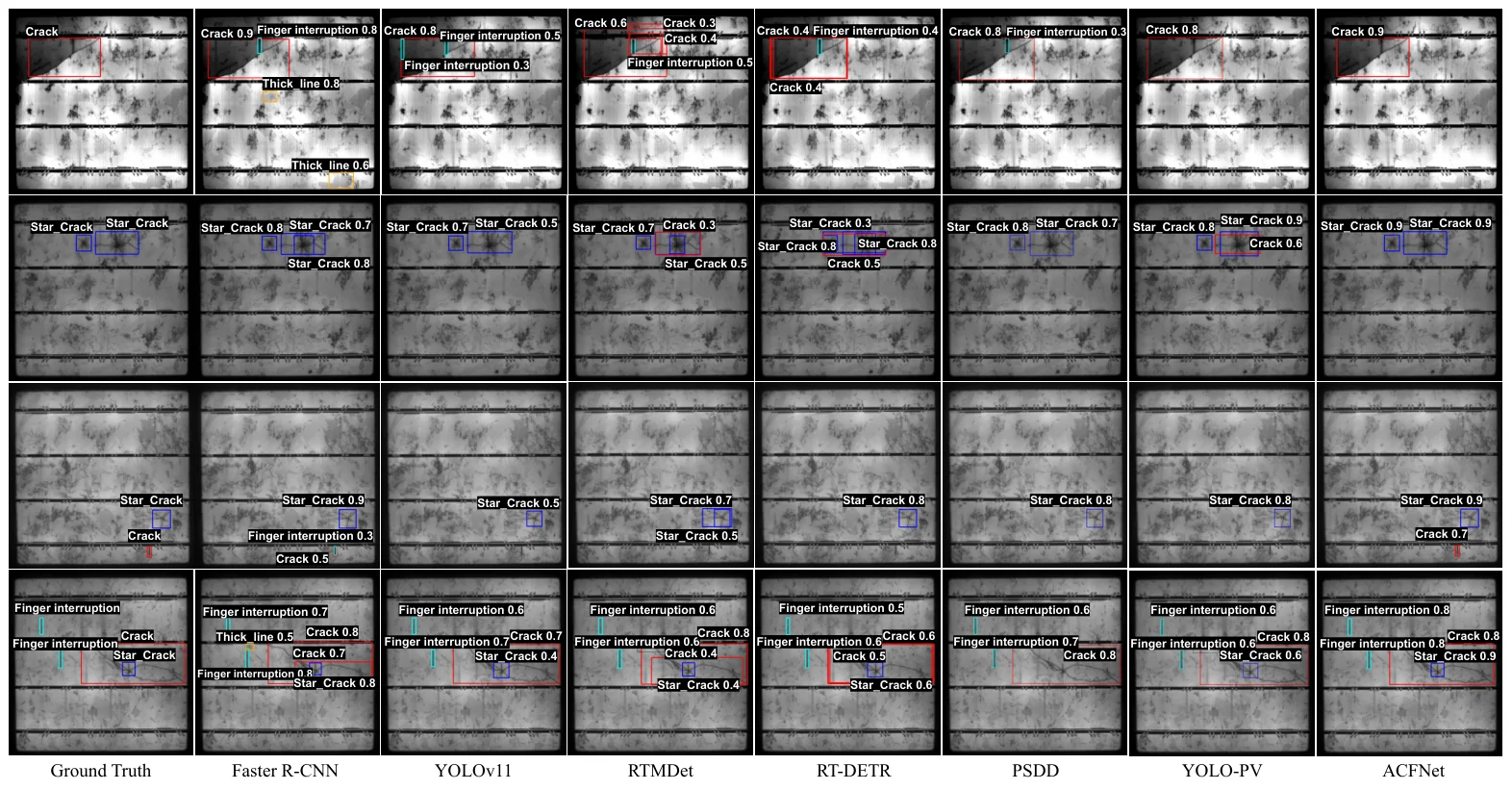
Qualitative comparison of defect detection results on the PVEL-AD dataset.

**Figure 7:**
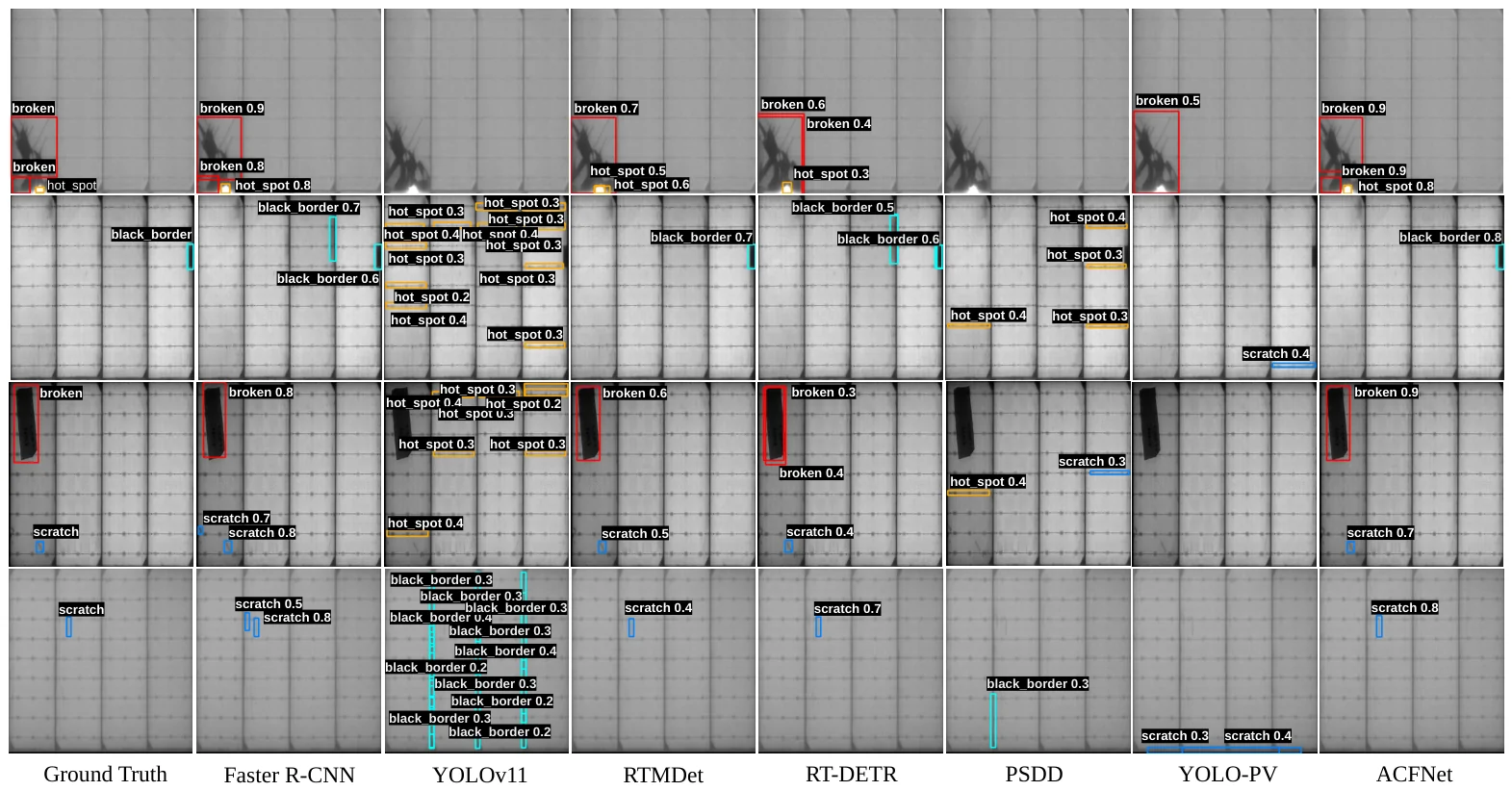
Qualitative comparison of defect detection results on the PV-Multi-Defect dataset.

To qualitatively evaluate the detection performance of ACFNet, we compare its visualization results with several representative methods, including Faster R-CNN [16], YOLOv11 [17], RTMDet [56], RT-DETR [18], PSDD [21], and YOLO-PV [29]. Figure 6 and Figure 7 present representative challenging scenarios from the PVEL-AD and PV-Multi-Defect datasets, respectively.

As shown in Figure 6, ACFNet demonstrates superior localization and classification accuracy on the PVEL-AD dataset. Specifically, for large cracks under complex background interference (row 1), Faster R-CNN, YOLOv11, RTMDet, and RT-DETR produce false-positive detections around the crack region, whereas ACFNet precisely bounds the entire defect. For adjacent star cracks (row 2), RTMDet, RT-DETR, and YOLO-PV incorrectly identify star cracks as crack defects, resulting in redundant detections, whereas ACFNet accurately distinguishes individual star-crack instances. For multiscale crack and star-crack defects (row 3), most competing methods fail to detect the small crack, whereas ACFNet successfully captures all target defects. For coexisting defects with discontinuous structures (row 4), Faster R-CNN and RTMDet output fragmented bounding boxes along the crack path, while PSDD misses the star crack. In contrast, ACFNet accurately detects all defects without fragmented predictions along the crack path.

From Figure 7, ACFNet consistently yields the most reliable detection results on the PV-Multi-Defect dataset. Specifically, for defects with significant scale variations (row 1), YOLOv11, PSDD, and YOLO-PV fail to detect the small hot spot. Under severe background clutter (row 2), most competing methods generate false-positive detections in non-defect regions. For coexisting heterogeneous defects (row 3), YOLOv11 and PSDD miss the scratch while producing background false positives, and YOLO-PV misses multiple targets. In the case of subtle defects with weak features (row 4), Faster R-CNN and other comparative methods suffer from frequent missed detections or false responses. In contrast, ACFNet robustly eliminates background interference and accurately localizes all target defects across these challenging scenarios.

#### 4.2.4. Running Efficiency and Operational Viability

As shown in Table 5, ACFNet contains only 3.0 M parameters and 7.1G FLOPs while achieving 75.7 FPS (13.2 ms latency) on an NVIDIA A100 GPU and 92.6 FPS (10.8 ms latency) on an NVIDIA RTX 4090 GPU. While ACFNet exhibits lower inference speed than some ultra-lightweight detectors such as YOLOv8 (156.9 FPS on A100) and YOLOX (83.3 FPS on A100), it remains faster than YOLOv12 (72.7 FPS on A100) and YOLO26 (64.8 FPS on A100), while substantially outperforming two-stage and Transformer-based models, such as Faster R-CNN (7.1 FPS) and Deformable DETR (4.9 FPS). Overall, ACFNet provides an effective trade-off between detection accuracy and computational cost. Reaching sub-15 ms latency across both test platforms (13.2 ms on A100 and 10.8 ms on RTX 4090) comfortably satisfies inline EL inspection throughput demands (>30 FPS). Natively supporting clean export to runtime acceleration engines (e.g., ONNX and TensorRT), ACFNet functions as a high-throughput software inference module that streams structured defect metadata to downstream Programmable Logic Controller (PLC) sorting hardware and Manufacturing Execution Systems (MES) for end-to-end process control and long-term quality traceability.

### 4.3. Ablation Experiments

#### 4.3.1. Structural Ablation

We perform a structural ablation to assess the effectiveness of each proposed component in ACFNet, as summarized in Table 6. All configurations were independently trained using five fixed random seeds under the same data split and training settings, and the results are reported as mean ± standard deviation. On PVEL-AD, introducing SCMB, ACF, or SCE individually increases the mean mAP@0.5 from 0.907 to 0.917, 0.915, and 0.916, respectively. Dual-module combinations achieve mean mAP@0.5 values of 0.922–0.925. On PV-Multi-Defect, the single-module configurations increase the mean mAP@0.5 from 0.847 to 0.852–0.853, while the dual-module configurations reach up to 0.866. Integrating all three modules achieves the highest mean mAP@0.5 on both datasets: 0.934 ± 0.003 on PVEL-AD and 0.876 ± 0.006 on PV-Multi-Defect.

**Figure 8:**
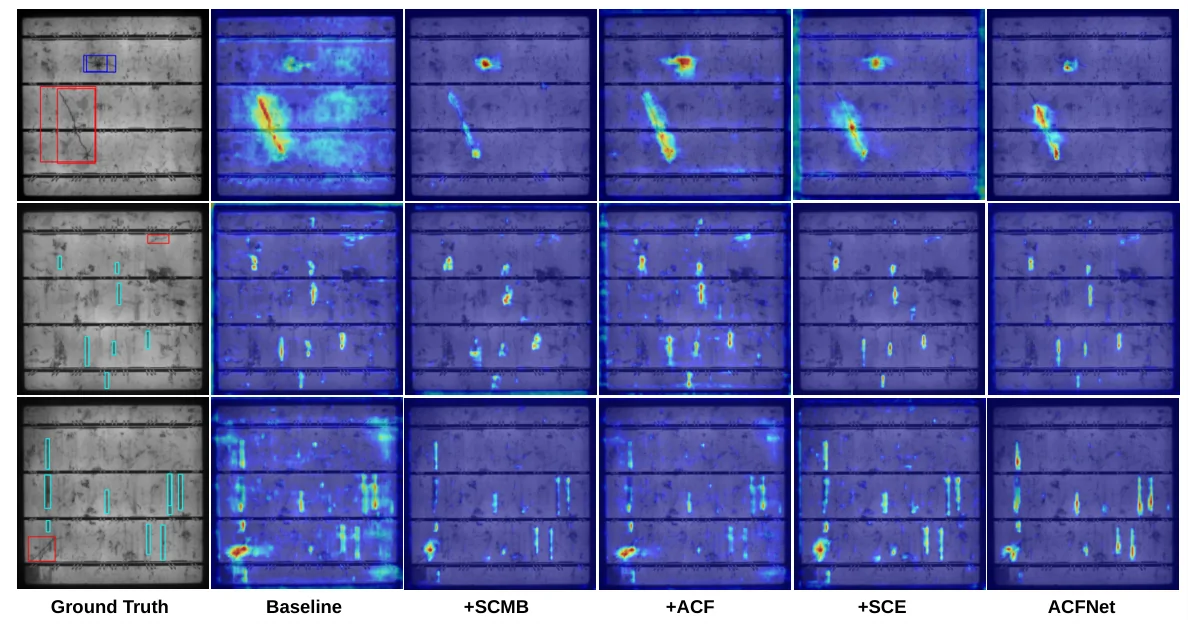
Grad-CAM visualizations of the ablation study on the PVEL-AD dataset.In the Ground Truth images, blue, red, and cyan boxes denote star_crack, crack, and finger_interruption defects, respectively.

**Figure 9:**
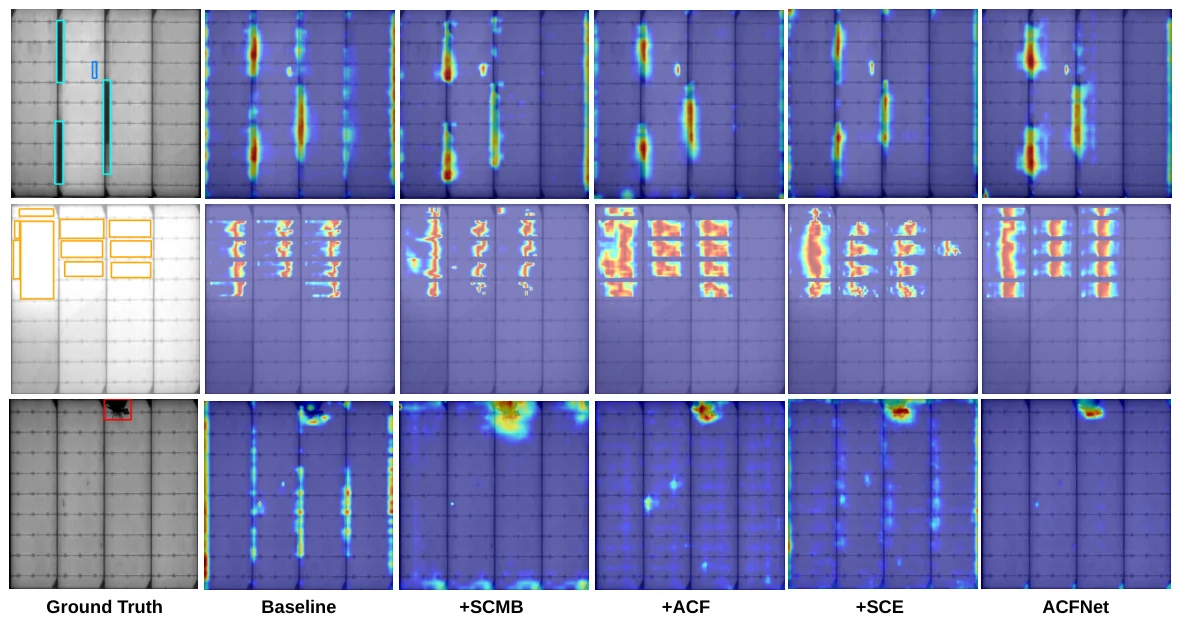
Grad-CAM visualizations of the ablation study on the PV-Multi-Defect dataset.In the Ground Truth images, red, cyan, blue, and orange boxes denote broken, black_border, scratch, and hot_spot defects, respectively.

The Grad-CAM visualizations in Figure 8 and Figure 9 were generated using feature layers at the three detection scales as target layers. The backpropagation target was the sum of maximum class confidence scores among the top 2% of predictions with confidence scores of at least 0.2. The activation maps were normalized to [0,1] and displayed using the JET color scale, where blue and red indicate low and high relative responses, respectively. The Baseline exhibits relatively diffuse activation responses with noticeable background activations. As the proposed modules are progressively integrated, the Grad-CAM responses become more concentrated on the annotated defect regions, with reduced activation in irrelevant background areas. The full ACFNet produces more compact and well-localized activation patterns, consistent with the quantitative results.

#### 4.3.2. Comparison with Related Modules

To further evaluate the effectiveness of the proposed modules, we compare SCMB, ACF, and SCE with the closely related ScConv, GFPN, and CBAM, respectively, under the same experimental settings. Each pair is evaluated on the same baseline, with only the corresponding module or feature-fusion structure changed. As shown in Table 7, SCMB, ACF, and SCE achieve higher mAP@0.5 and mAP@0.5:0.95 than their corresponding comparison modules on both PVEL-AD and PV-Multi-Defect. Although Precision and Recall exhibit different trade-offs, the consistent improvements in both mAP metrics support the effectiveness of the proposed structural designs.

#### 4.3.3. Sub-Component Ablation

To validate the detailed structural choices within each proposed module, we perform further sub-component ablation studies on the PVEL-AD and PV-Multi-Defect datasets.

**SCMB:** As shown in Table 8, individual evaluations of SMU and CMU of SCMB confirm their complementary roles in feature enhancement. On PVEL-AD, introducing SMU or CMU individually increases the mAP@0.5 from 0.906 to 0.912 and 0.914, respectively, with SMU achieving the highest recall (0.882). Jointly integrating both units yields the best performance, boosting the mAP@0.5 to 0.918, precision to 0.873, and F1-score to 0.863. A consistent trend is observed on PV-Multi-Defect, where combining SMU and CMU achieves optimal overall results, elevating the mAP@0.5 to 0.856 while reaching peak recall (0.825) and F1-score (0.828).
**ACF:** To evaluate the impact of different cross-layer connections in ACF, we conduct ablation studies on Path1 ($f_{4}^{\prime }\to f_{5}^{s_{1}}$), Path2 ($f_{3}^{\prime }\to f_{4}^{s_{1}}$), and Path3 ($f_{4}^{s_{1}}\to f_{5}^{s_{2}}$), as illustrated in Figure 2b. As reported in Table 9, removing any cross-layer connection causes performance degradation compared to the full ACF configuration. On PVEL-AD, the complete three-path ACF achieves the optimal trade-off with the highest mAP@0.5 (0.915) and F1-score (0.868), boosting performance over the Baseline (0.906). On PV-Multi-Defect, the full ACF similarly obtains the peak mAP@0.5 of 0.855, yielding higher mAP@0.5 than the incomplete path variants. These results demonstrate that all three asymmetric paths are essential for effective multiscale feature interaction.
**SCE:** Table 10 summarizes the detection performance under different combinations of MS, SA, and CA within the SCE module. On PVEL-AD, while partial configurations like MS + SA and SA + CA emphasize precision (0.871) and recall (0.877) respectively, integrating all three components yields the best overall performance, boosting mAP@0.5 to 0.917, recall to 0.880, and F1-score to 0.873. On PV-Multi-Defect, the complete SCE similarly achieves the peak mAP@0.5 of 0.849 and highest recall of 0.794, outperforming partial combinations and the Baseline. These results confirm the joint synergy among MS, SA, and CA in effectively capturing weak defect patterns.

### 4.4. Generalization Experiment

To evaluate the cross-domain generalization capability of ACFNet, experiments are conducted on the NEU-Det dataset [62], a widely used benchmark for steel surface defect detection. The dataset contains six representative defect categories: Crazing (Cr), Inclusion (In), Patches (Pa), Pitted Surface (Ps), Rolled-in Scale (Rs), and Scratches (Sc). We compare ACFNet with eleven representative object detection models, including one-stage, two-stage, and Transformer-based detectors.

Table 11 presents the comparison results on the NEU-Det dataset. ACFNet achieves the highest mAP of 0.801, outperforming all competing methods. Compared with Deformable DETR, YOLOv11, and YOLO26, ACFNet improves the mAP by 0.020, 0.035, and 0.026, respectively. Moreover, ACFNet achieves the highest AP on Cr (0.542), Pa (0.942), and Sc (0.965), exceeding the second-best results by 0.008, 0.028, and 0.012, respectively. Although several competing methods achieve slightly higher AP on individual categories, ACFNet achieves the highest overall mAP, indicating strong cross-domain generalization capability.

### 4.5. Robustness Analysis

We evaluate robustness on PVEL-AD and PV-Multi-Defect under eight corruption types: Gaussian noise, shot noise, impulse noise, snow, fog, brightness, contrast, and pixelation. ACFNet is compared with YOLOv11, DINO, RT-DETR, and three PV defect detection models: YOLO-PV, AE-YOLO, and PSDD. All models use fixed trained weights and are evaluated under the same corruption settings within each dataset without retraining. P denotes clean-validation mAP@0.5, mPC is the mean mAP@0.5 across 40 settings (eight types with five parameter settings each), and rPC = mPC/P. As shown in Table 12, ACFNet achieves the highest mPC on both datasets, with values of 0.612 and 0.470, respectively. It also achieves the highest rPC of 0.656 on PVEL-AD. On PV-Multi-Defect, its rPC is 0.532, compared with the highest value of 0.553 achieved by PSDD. These results indicate that ACFNet exhibits good overall robustness under the evaluated synthetic image corruptions.

## 5. Conclusions

In this paper, we propose ACFNet, a novel single-stage object detection framework for photovoltaic defect detection. The framework integrates three key components: an SCMB to reduce feature redundancy and suppress complex background noise, an ACF strategy to handle extreme scale variations via asymmetric cross-layer connections, and an SCE to enhance the contextual representation of defects with discontinuous structures. Extensive experiments on the PVEL-AD and PV-Multi-Defect datasets demonstrate the effectiveness of the proposed method. ACFNet achieves strong overall detection performance across both datasets and maintains competitive performance across different defect categories. On PV-Multi-Defect, although some competing methods achieve higher AP for hot spot, scratch, and no-electricity, ACFNet demonstrates strong overall performance and a favorable balance across categories. The proposed method also shows strong performance on crack and star crack defects.

Although ACFNet achieves superior detection performance, the introduction of the proposed modules inevitably increases computational complexity, and some defect categories with weak visual characteristics, such as hot spots and scratches, remain challenging to detect. Future work will focus on developing lighter feature enhancement strategies to reduce computational overhead, facilitating edge-hardware deployment and integration with automated pass/reject sorting systems in high-throughput PV manufacturing lines. Additionally, we will explore more discriminative feature representations to improve the detection of subtle and irregular defects in photovoltaic electroluminescence images.

## Figures and Tables

**Table 1:** Performance comparison of multiple models on the PVEL-AD dataset.

Model	mAP@0.5	mAP@0.5:0.95	P	R	F1	AP@0.5							AP@0.5:0.95						
						Bc	Cr	Fi	Hd	Sci	Scr	Tl	Bc	Cr	Fi	Hd	Sci	Scr	Tl
RetinaNet [49]	0.892	0.661	0.857	0.816	0.836	0.987	0.751	0.804	0.995	0.994	0.838	0.872	0.933	0.467	0.432	0.688	0.968	0.642	0.494
FCOS [50]	0.851	0.617	0.738	0.688	0.712	0.993	0.763	0.821	0.645	0.995	0.837	0.905	0.942	0.471	0.455	0.373	0.959	0.613	0.509
ATSS [51]	0.910	0.665	0.864	0.839	0.851	**0.999**	0.781	0.871	**0.997**	0.995	0.827	0.899	0.950	0.475	0.478	0.670	0.973	0.602	**0.510**
YOLOv8 [52]	0.905	0.652	0.899	0.852	0.875	0.981	0.775	0.918	0.985	0.985	0.851	0.840	0.955	0.506	**0.540**	0.468	0.968	0.645	0.485
YOLOv11 [17]	0.906	0.639	0.863	0.859	0.861	0.991	0.782	0.910	0.993	0.986	0.795	0.886	0.958	0.494	0.521	0.472	0.960	0.579	0.490
YOLOv12 [53]	0.912	0.643	0.878	0.847	0.862	0.989	0.786	0.902	0.995	0.984	0.846	0.883	0.956	0.508	0.519	0.470	0.941	0.622	0.489
YOLO26 [54]	0.906	0.653	0.877	0.847	0.862	0.988	0.739	0.906	0.995	0.990	0.856	0.871	0.959	0.505	0.520	0.490	0.967	0.648	0.480
YOLOX [55]	0.902	0.649	0.800	0.789	0.794	0.973	0.750	0.873	0.996	0.993	0.893	0.837	0.881	0.461	0.486	0.696	0.955	0.599	0.463
Faster R-CNN [16]	0.760	0.558	0.690	0.766	0.726	0.989	0.781	0.893	–	0.992	0.787	0.881	0.931	0.462	0.497	−	0.968	0.574	0.472
Cascade R-CNN [47]	0.746	0.561	0.714	0.735	0.724	0.980	0.747	0.873	–	0.995	0.798	0.830	0.930	0.469	0.497	−	0.966	0.595	0.469
Dynamic R-CNN [48]	0.765	0.565	0.714	0.747	0.730	0.980	0.795	0.882	–	**0.997**	0.833	0.866	0.921	0.476	0.507	−	0.963	0.608	0.480
DETR [57]	0.778	0.499	0.715	0.730	0.722	0.990	0.644	0.534	0.957	0.992	0.579	0.748	0.938	0.292	0.203	0.462	**0.982**	0.311	0.303
Deformable DETR [58]	0.877	0.635	0.873	0.833	0.853	0.983	0.730	0.831	0.985	0.993	0.737	0.878	0.923	0.396	0.460	0.783	0.963	0.442	0.479
Conditional DETR [59]	0.912	**0.670**	0.896	0.882	0.889	0.998	0.804	0.872	0.995	0.994	0.839	0.884	0.925	0.493	0.437	0.772	0.974	0.609	0.482
DAB-DETR [60]	0.905	0.667	0.871	0.858	0.864	0.988	0.793	0.863	0.996	0.995	0.801	0.902	0.929	0.455	0.470	**0.788**	0.968	0.577	0.485
DINO [61]	0.906	0.653	0.875	0.862	0.868	0.989	0.798	0.883	0.987	0.995	0.790	0.898	0.948	0.511	0.534	0.471	0.966	0.648	0.495
RTMDet [56]	0.913	0.664	0.850	0.881	0.865	0.992	0.751	0.904	0.995	**0.997**	0.860	0.892	0.923	0.477	0.509	0.729	0.917	0.601	0.494
RT-DETR [18]	0.911	0.668	0.896	0.865	0.880	0.987	0.758	0.913	0.995	0.995	0.847	0.881	0.937	0.478	0.539	0.683	0.919	0.622	0.498
PSDD [21]	0.924	0.657	0.882	0.842	0.862	0.989	0.789	0.919	0.992	0.991	0.901	0.886	0.953	0.500	0.524	0.510	0.970	0.648	0.490
AE-YOLO [13]	0.917	0.644	0.864	0.835	0.849	0.989	0.768	0.914	0.995	0.984	0.898	0.874	0.953	0.476	0.521	0.480	0.930	0.649	0.498
YOLO-PV [29]	0.912	0.650	0.863	0.886	0.874	0.993	0.762	0.916	0.992	0.992	0.881	0.846	0.959	0.495	0.534	0.469	0.968	0.649	0.471
**ACFNet (Ours)**	**0.933**	0.652	**0.907**	**0.890**	**0.898**	0.991	**0.821**	**0.921**	0.995	0.990	**0.907**	**0.907**	**0.960**	**0.525**	0.525	0.465	0.930	**0.650**	**0.510**

Note: The best results are highlighted in bold, and ‘−’ indicates unavailable results.

**Table 2:** Performance comparison of multiple models on the PV-Multi-Defect dataset.

Model	mAP@0.5	mAP@0.5:0.95	P	R	F1	AP@0.5					AP@0.5:0.95				
						Br	Hs	Bb	Sc	Ne	Br	Hs	Bb	Sc	Ne
RetinaNet [49]	0.829	0.561	0.735	0.768	0.751	0.771	**0.935**	0.755	0.689	**0.996**	0.555	0.659	0.314	0.281	**0.995**
FCOS [50]	0.841	0.567	0.822	0.781	0.801	0.785	0.914	0.745	**0.773**	0.987	0.586	0.641	0.320	**0.311**	0.977
ATSS [51]	0.815	0.548	0.740	0.775	0.757	0.692	0.930	0.761	0.707	0.985	0.457	0.659	0.344	0.291	0.990
YOLOv8 [52]	0.842	0.552	0.808	0.809	0.808	0.891	0.827	0.955	0.669	0.867	0.604	0.593	0.488	0.240	0.836
YOLOv11 [17]	0.844	0.544	0.838	0.735	0.783	0.831	0.898	0.954	0.681	0.857	0.514	0.578	0.536	0.241	0.850
YOLOv12 [53]	0.808	0.551	0.751	0.765	0.758	0.692	0.819	0.960	0.683	0.883	0.550	0.578	0.518	0.249	0.859
YOLO26 [54]	0.787	0.534	0.865	0.715	0.783	0.802	0.795	0.880	0.549	0.910	0.504	0.562	0.524	0.210	0.869
YOLOX [55]	0.836	0.527	0.801	0.751	0.775	0.841	0.911	0.778	0.658	0.993	0.489	0.645	0.329	0.254	0.917
Faster R-CNN [16]	0.841	0.582	0.857	0.816	0.836	0.816	0.928	0.754	0.714	0.995	0.641	0.650	0.351	0.286	0.983
Cascade R-CNN [47]	0.840	0.579	0.847	0.805	0.825	0.825	0.932	0.774	0.676	0.994	0.590	**0.662**	0.385	0.283	0.975
Dynamic R-CNN [48]	0.849	0.585	0.757	0.795	0.776	0.883	0.917	0.744	0.719	0.981	0.623	0.648	0.372	0.299	0.981
DETR [57]	0.799	0.473	0.692	0.762	0.725	0.816	0.912	0.756	0.516	0.995	0.460	0.529	0.274	0.118	0.985
Deformable DETR [58]	0.847	0.573	0.857	0.734	0.791	0.843	**0.935**	0.747	0.724	0.987	0.612	0.652	0.326	0.296	0.977
Conditional DETR [59]	0.838	0.565	0.858	0.786	0.820	0.860	0.906	0.713	0.734	0.976	0.589	0.624	0.361	0.265	0.985
DAB-DETR [60]	0.836	0.573	0.870	0.779	0.822	0.841	0.929	0.700	0.723	0.986	0.589	0.638	0.368	0.275	0.994
DINO [61]	0.860	0.581	0.859	0.727	0.787	0.892	0.835	0.851	0.742	0.981	0.630	0.632	0.559	0.232	0.853
RTMDet [56]	0.842	0.584	0.814	0.742	0.776	0.765	0.867	0.833	0.762	0.984	0.655	0.584	0.406	0.290	0.986
RT-DETR [18]	0.768	0.505	0.750	0.664	0.705	0.702	0.753	0.893	0.656	0.837	0.490	0.539	0.528	0.241	0.729
PSDD [21]	0.830	0.551	0.750	0.781	0.765	0.754	0.860	0.978	0.662	0.898	0.517	0.575	0.535	0.260	0.869
AE-YOLO [13]	0.826	0.575	0.863	0.750	0.803	0.838	0.797	0.964	0.653	0.878	0.630	0.592	0.556	0.246	0.853
YOLO-PV [29]	0.847	0.568	0.840	0.794	0.816	0.917	0.828	0.918	0.683	0.889	0.641	0.570	0.547	0.256	0.824
**ACFNet (Ours)**	**0.884**	**0.598**	**0.878**	**0.822**	**0.849**	**0.922**	0.861	**0.987**	0.717	0.934	**0.698**	0.625	**0.560**	0.255	0.852

Note: The best results are highlighted in bold.

**Table 3:** Performance comparison over five fixed random seeds on the PVEL-AD and PV-Multi-Defect datasets.

Dataset	Model	mAP@0.5	mAP@0.5:0.95	P	R	F1
PVEL-AD	PSDD	0.919±0.004	0.652±0.015	0.857±0.052	0.864±0.039	0.858±0.016
	**ACFNet (Ours)**	0.934±0.003 *	0.647±0.006	0.890±0.015	0.885±0.012	0.887±0.005
PV-Multi-Defect	DINO	0.861±0.006	0.582±0.005	0.846±0.016	0.766±0.047	0.804±0.030
	**ACFNet (Ours)**	0.876±0.006	0.591±0.015	0.834±0.027	0.823±0.016	0.828±0.013

Note: Results are reported as mean ± standard deviation over five fixed random seeds. The best result for each metric within the same dataset is highlighted in bold. * indicates a statistically significant improvement over PSDD on PVEL-AD (paired *t*-test, p<0.05).

**Table 4:** Paired bootstrap comparison of mAP@0.5 on the two test sets, where ΔmAP@0.5 denotes ACFNet minus the corresponding competitor.

Dataset	Comparison	ΔmAP@0.5	95% Paired Bootstrap CI
PVEL-AD	ACFNet vs. PSDD	+0.015	[0.002,0.029]
PV-Multi-Defect	ACFNet vs. DINO	+0.015	[−0.015,0.046]

**Table 5:** Comparison of computational complexity and inference speed on NVIDIA A100 and NVIDIA RTX 4090 GPUs.

Model	FLOPs (G)	Params (M)	NVIDIA A100		NVIDIA RTX 4090	
			Latency (ms)	FPS	Latency (ms)	FPS
RetinaNet [49]	82.5	36.5	26.0	38.5	14.7	67.8
FCOS [50]	78.7	32.1	29.3	34.1	16.4	61.1
ATSS [51]	80.6	32.1	26.0	38.4	16.0	62.5
YOLOv8 [52]	8.1	3.0	**6.4**	**156.9**	**6.0**	**165.8**
YOLOv11 [17]	6.3	2.6	8.6	116.1	8.0	125.6
YOLOv12 [53]	6.3	2.6	13.8	72.7	11.6	85.9
YOLO26 [54]	5.2	2.3	15.4	64.8	11.9	84.0
YOLOX [55]	7.6	5.0	12.0	83.3	7.7	130.2
Faster R-CNN [16]	90.9	41.3	140.8	7.1	28.0	35.7
Cascade R-CNN [47]	119.0	69.2	243.9	4.1	35.6	28.1
Dynamic R-CNN [48]	90.9	41.4	169.5	5.9	27.5	36.3
DETR [57]	38.8	41.6	42.9	23.3	17.3	57.7
Deformable DETR [58]	79.6	40.1	204.1	4.9	40.8	24.5
Conditional DETR [59]	42.1	43.5	66.2	15.1	21.0	47.6
DAB-DETR [60]	43.1	43.7	71.4	14.0	25.4	39.3
DINO [61]	119.0	47.5	294.1	3.4	61.3	16.3
RTMDet [56]	8.0	4.9	35.0	28.6	21.7	46.0
RT-DETR [18]	57.0	19.9	50.8	19.7	21.2	47.2
PSDD [21]	10.6	4.0	9.9	101.0	7.7	129.9
AE-YOLO [13]	**5.1**	**2.1**	10.1	99.1	9.6	104.4
YOLO-PV [29]	12.8	5.5	10.1	98.6	9.7	102.7
**ACFNet (Ours)**	7.1	3.0	13.2	75.7	10.8	92.6

Note: FLOPs, floating-point operations; Params, parameters; FPS, frames per second. The best results are highlighted in bold.

**Table 6:** Ablation study of the proposed components on PVEL-AD and PV-Multi-Defect datasets.

Dataset	Module	mAP@0.5	mAP@0.5:0.95	P	R	F1
PVEL-AD	Baseline	0.907±0.006	0.641±0.012	0.865±0.029	0.851±0.013	0.858±0.012
	SCMB	0.917±0.004	0.644±0.008	0.869±0.032	0.860±0.012	0.864±0.011
	ACF	0.915±0.005	0.657±0.020	0.857±0.005	0.878±0.007	0.867±0.004
	SCE	0.916±0.006	0.643±0.008	0.861±0.006	0.848±0.030	0.854±0.018
	SCMB + ACF	0.923±0.006	0.648±0.005	0.884±0.018	0.862±0.004	0.873±0.011
	ACF + SCE	0.925±0.007	0.645±0.010	0.886±0.014	0.867±0.020	0.876±0.010
	SCMB + SCE	0.922±0.009	0.643±0.011	0.855±0.046	0.864±0.017	0.859±0.025
	Ours (Full)	0.934±0.003	0.647±0.006	0.890±0.015	0.885±0.012	0.887±0.005
PV-Multi-Defect	Baseline	0.847±0.005	0.564±0.016	0.817±0.021	0.774±0.025	0.795±0.009
	SCMB	0.853±0.014	0.589±0.016	0.844±0.032	0.794±0.040	0.817±0.010
	ACF	0.853±0.006	0.590±0.009	0.819±0.021	0.793±0.011	0.806±0.010
	SCE	0.852±0.004	0.578±0.012	0.797±0.053	0.783±0.010	0.789±0.023
	SCMB + ACF	0.855±0.008	0.578±0.017	0.836±0.031	0.789±0.043	0.811±0.009
	ACF + SCE	0.866±0.003	0.581±0.014	0.835±0.019	0.800±0.015	0.817±0.006
	SCMB + SCE	0.863±0.009	0.594±0.008	0.822±0.014	0.815±0.023	0.818±0.008
	Ours (Full)	0.876±0.006	0.591±0.015	0.834±0.027	0.823±0.016	0.828±0.013

Note: The best results are highlighted in bold.

**Table 7:** Comparison of SCMB, ACF, and SCE with related existing modules on the PVEL-AD and PV-Multi-Defect datasets.

Method	PVEL-AD					PV-Multi-Defect				
	mAP@0.5	mAP@0.5:0.95	P	R	F1	mAP@0.5	mAP@0.5:0.95	P	R	F1
ScConv [35]	0.909	0.628	0.855	0.822	0.838	0.846	0.599	0.796	0.793	0.794
SCMB (Ours)	0.918	0.642	0.873	0.853	0.863	0.856	0.610	0.831	0.825	0.828
GFPN [28]	0.897	0.623	0.854	0.857	0.855	0.843	0.570	0.752	0.828	0.788
ACF (Ours)	0.915	0.647	0.862	0.875	0.868	0.855	0.582	0.824	0.804	0.814
CBAM [37]	0.904	0.634	0.882	0.862	0.872	0.837	0.559	0.786	0.754	0.770
SCE (Ours)	0.917	0.642	0.866	0.880	0.873	0.849	0.577	0.747	0.794	0.770

**Table 8:** Ablation study of the SMU and CMU components in SCMB on the PVEL-AD and PV-Multi-Defect datasets.

SMU	CMU	PVEL-AD				PV-Multi-Defect			
		mAP@0.5	P	R	F1	mAP@0.5	P	R	F1
-	-	0.906	0.863	0.859	0.861	0.844	0.838	0.735	0.783
✓	-	0.912	0.819	0.882	0.849	0.850	0.861	0.784	0.821
-	✓	0.914	0.821	0.871	0.845	0.853	0.866	0.764	0.812
✓	✓	0.918	0.873	0.853	0.863	0.856	0.831	0.825	0.828

Note: The symbol “✓” indicates the inclusion of the corresponding module.

**Table 9:** Ablation study of different cross-layer connections in ACF on the PVEL-AD and PV-Multi-Defect datasets.

Path1	Path2	Path3	PVEL-AD				PV-Multi-Defect			
			mAP@0.5	P	R	F1	mAP@0.5	P	R	F1
-	-	-	0.906	0.863	0.859	0.861	0.844	0.838	0.735	0.783
✓	✓	-	0.912	0.850	0.875	0.862	0.852	0.787	0.818	0.802
✓	-	✓	0.913	0.847	0.868	0.857	0.828	0.819	0.756	0.786
-	✓	✓	0.912	0.836	0.890	0.862	0.841	0.803	0.809	0.806
✓	✓	✓	0.915	0.862	0.875	0.868	0.855	0.824	0.804	0.814

Note: The symbol “✓” indicates the inclusion of the corresponding module.

**Table 10:** Component ablation of SCE on the PVEL-AD and PV-Multi-Defect datasets.

MS	SA	CA	PVEL-AD				PV-Multi-Defect			
			mAP@0.5	P	R	F1	mAP@0.5	P	R	F1
-	-	-	0.906	0.863	0.859	0.861	0.844	0.838	0.735	0.783
✓	✓	-	0.914	0.871	0.869	0.870	0.839	0.797	0.790	0.794
✓	-	✓	0.913	0.864	0.850	0.857	0.843	0.843	0.790	0.816
-	✓	✓	0.912	0.858	0.877	0.867	0.845	0.838	0.772	0.804
✓	✓	✓	0.917	0.866	0.880	0.873	0.849	0.747	0.794	0.770

Note: The symbol “✓” indicates the inclusion of the corresponding module.

**Table 11:** Performance comparison on the NEU-Det dataset.

Model	mAP@0.5	P	R	F1	AP@0.5					
					Cr	In	Pa	Ps	Rs	Sc
FCOS [50]	0.735	0.766	0.668	0.714	0.492	0.794	0.897	0.752	0.588	0.887
YOLOv8 [52]	0.773	0.710	0.742	0.726	0.483	0.816	0.914	0.803	0.667	0.953
YOLOv11 [17]	0.766	0.745	0.710	0.727	0.393	**0.873**	0.892	0.762	0.733	0.945
YOLOv12 [53]	0.756	0.729	0.706	0.717	0.503	0.823	0.888	0.747	0.666	0.906
YOLOX [55]	0.683	0.712	0.613	0.659	0.246	0.747	0.900	0.637	0.663	0.906
YOLO26 [54]	0.775	0.801	0.704	0.749	0.534	0.856	0.864	0.790	0.656	0.952
Faster R-CNN [16]	0.732	0.773	0.724	0.748	0.453	0.813	0.893	0.714	0.568	0.951
Dynamic R-CNN [48]	0.755	0.700	0.726	0.713	0.467	0.812	0.913	0.720	0.698	0.919
Cascade R-CNN [47]	0.754	0.739	0.724	0.731	0.488	0.814	0.882	0.758	0.672	0.948
Deformable DETR [58]	0.781	0.798	**0.754**	**0.775**	0.496	0.849	0.880	0.791	**0.754**	0.917
RT-DETR [18]	0.706	0.699	0.614	0.654	0.336	0.735	0.880	**0.816**	0.553	0.915
ACFNet (ours)	**0.801**	**0.815**	0.727	0.769	**0.542**	0.844	**0.942**	0.804	0.708	**0.965**

Note: The best results are highlighted in bold.

**Table 12:** Robustness evaluation under eight corruption conditions on PVEL-AD and PV-Multi-Defect.

Model	PVEL-AD			PV-Multi-Defect		
	P	mPC	rPC	P	mPC	rPC
YOLOv11 [17]	0.906	0.570	0.629	0.844	0.460	0.545
DINO [61]	0.906	0.588	0.649	0.860	0.468	0.544
RT-DETR [18]	0.911	0.560	0.615	0.768	0.408	0.531
PSDD [21]	0.924	0.594	0.643	0.830	0.459	**0.553**
AE-YOLO [13]	0.917	0.588	0.641	0.826	0.441	0.534
YOLO-PV [29]	0.912	0.575	0.630	0.847	0.467	0.551
**ACFNet (Ours)**	**0.933**	**0.612**	**0.656**	**0.884**	**0.470**	0.532

Note: The best result for each metric within each dataset is highlighted in bold.

## Data Availability

Our code is available at https://github.com/kaikaichen66/ACFNet, accessed on 5 August 2026 (commit 070edf6), and the datasets used in this study are publicly available at https://github.com/binyisu/PVEL-AD, accessed on 15 March 2026 and https://github.com/CCNUZFW/PV-Multi-Defect, accessed on 15 March 2026. Reproduction instructions are provided in the repository.
